# Comparison of bacterial community structure and potential functions in hypoxic and non-hypoxic zones of the Changjiang Estuary

**DOI:** 10.1371/journal.pone.0217431

**Published:** 2019-06-06

**Authors:** Dong-Mei Wu, Qiu-Ping Dai, Xue-Zhu Liu, Ying-Ping Fan, Jian-Xin Wang

**Affiliations:** 1 Marine Microorganism Ecological & Application Lab, Zhejiang Ocean University, Zhejiang, China; 2 Donghai Science and Technology College, Zhejiang Ocean University, Zhejiang, China; Stazione Zoologica Anton Dohrn, ITALY

## Abstract

Bacterioplankton play a key role in the global cycling of elements. To characterize the effects of hypoxia on bacterioplankton, bacterial community structure and function were investigated in the Changjiang Estuary. Water samples were collected from three layers (surface, middle, and bottom) at ten sampling sites in the Changjiang Estuary hypoxic and non-hypoxic zones. The community structure was analyzed using high-throughput sequencing of 16S rDNA genes, and the predictive metagenomic approach was used to investigate the functions of the bacterial community. Co-occurrence networks are constructed to investigate the relationship between different bacterioplankton. The results showed that community composition in hypoxic and non-hypoxic zones were markedly different. The diversity and richness of bacterial communities in the bottom layer (hypoxic zone) were remarkably higher than that of the surface layer (non-hypoxic). In the non-hypoxic zone, it was found that Proteobacteria, Bacteroidetes, and Flavobacteriia were the dominant groups while Alphaproteobacteria, SAR406 and Deltaproteobacteria were the dominant groups in the hypoxic zone. From the RDA analysis, it was shown that dissolved oxygen (DO) explained most of the bacterial community variation in the redundancy analysis targeting only hypoxia zones, whereas nutrients and salinity explained most of the variation across all samples in the Changjiang Estuary. To understand the genes involved in nitrogen metabolism, an analysis of the oxidation state of nitrogen was performed. The results showed that the bacterial community in the surface layer (non-hypoxic) had more genes involved in dissimilatory nitrate reduction, assimilatory nitrate reduction, denitrification, and anammox, while that in the middle and bottom layers (hypoxic zone) had more abundant genes associated with nitrogen fixation and nitrification. Co-occurrence networks revealed that microbial assemblages in the middle and bottom layers shared more niche spaces than in the surface layer (non-hypoxic zone). The environmental heterogeneity in the hypoxic and non-hypoxic zones might be important environmental factors that determine the bacterial composition in these two zones.

## Introduction

Bacterioplankton are important microorganisms in marine ecosystems, that play important roles in biogeochemical cycling, energy flow, and the ocean food web. Bacterioplankton in aquatic ecosystems are very sensitive to changes in environmental conditions, and thus bacterial community composition can act as an environmental indicator[1]. Studies of the relationships between physicochemical factors and bacterial community structures can enhance our understanding of microbial ecology. Additionally, investigations of functional potential of bacteria will assist in understanding their roles in biogeochemical cycling.

Hypoxia is a common phenomenon in many estuarine regions, and it causes a deterioration in the structure and function of ecosystems. Previous studies that have investigated hypoxia have been conducted on the global ocean scale. These studies have examined the causes of hypoxia, and the impact on the sustainability of the ecosystem. The Changjiang Estuary is located at the mouth of the Changjiang River[2]. This region is extremely complicated and dynamic because of the mixture of water from the Changjiang River exchanged with water from the Taiwan Warm Current (TWC). Previous investigations have indicated that the Changjiang Estuary has been suffering from eutrophication and seasonal hypoxia. These issues may be caused by water stratification and decomposition of organic matter that comes from upstream sediments and dissolved organic compounds [3]. Dissolved oxygen (DO) plays key role in altering bacterial communities in many marine and fresh water systems[4, 5]. In addition, a low DO concentration could inhibit the growth of aerobic organisms, and alter the food chain structure[6]. All of these conditions may lead to changes in biogeochemical cycling [7].

Previous bacterial community studies have been conducted in this area[8, 9]. Liu *et al*. used PCR-denaturing gradient gel electrophoresis (DGGE) to analyze the bacterial community composition in hypoxic area of the Changjiang Estuary and found that Proteobacteria, Bacteroides, Firmicutes, and Cyanobacteria dominated the hypoxic zone [10]. A previous study that investigated the temporal distribution of the bacterial community structure in the Changjiang Estuary hypoxic area and the adjacent East China Sea found that Gammaproteobacteria, Cytophaga–Flavobacteria–Bacteroides (CFB), Deltaproteobacteria, Cyanobacteria, and Firmicutes were the dominant groups in the hypoxic area. This study also showed that dissolved organic carbon (DOC) had a significant influence on the bacterial community[8]. However, there is relatively little information regarding the functions of the bacterial populations in the hypoxic and non-hypoxic zones of the Changjiang Estuary.

In this study, high-throughput sequencing technology is used to target 16S rDNA genes to analyze bacterial diversity. In addition, metagenomic analysis is used to compare microbial functions of different water layers in the Changjiang Estuary hypoxic area. This research provides insights into the relationship between hypoxia and bacterial diversity and biological functions.

## Materials and methods

### Sampling areas and sampling

**Fig 1:**
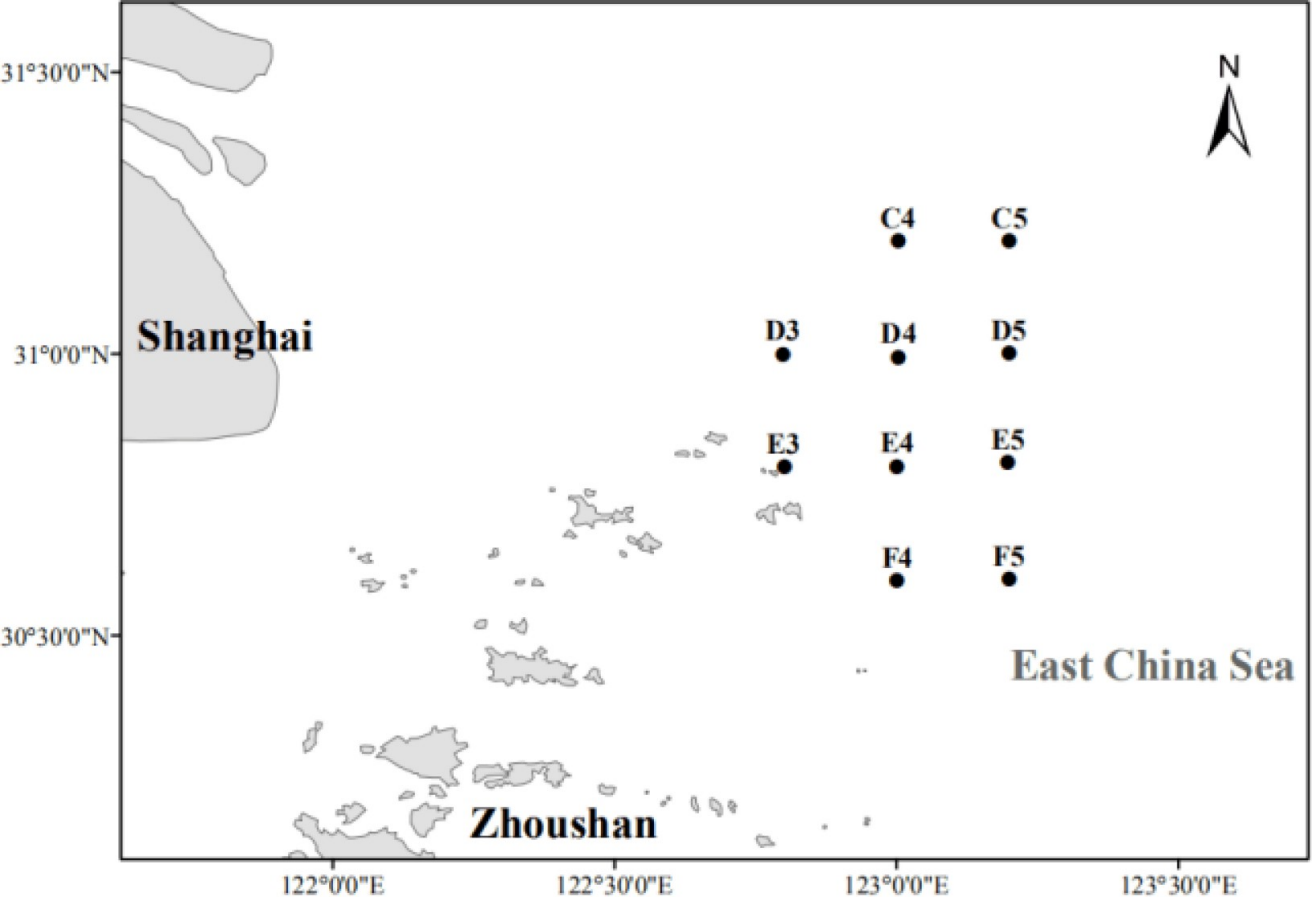
Sampling sites. Map showing the location of sampling sites in the Changjiang Estuary.

In July 2016, water samples were collected using the SBE 32 sampler (Sea-Bird Electronics, Washington, USA) from ten hypoxic and non-hypoxic sites in the Changjiang Estuary (Fig 1). No specific permissions were required for these locations and activities. Table 1 lists the latitude and longitude of the sampling sites. Samples from each site were collected from the surface layer (10 m depth), middle layer (20 m depth), and bottom layer (40–55 m depth), and the samples are designated as S (surface), M (middle), B (bottom) in the following analysis. Samples were collected for DNA isolation using a vacuum pump suction filter under 20 kPa. One liter of seawater per sample was filtered, first through a 3-μm pore size polycarbonate nucleopore membrane then through a 0.22-μm membrane(Merck Millipore Ltd., USA). The 0.22-μm filters were preserved in 5-ml sterile cryopreservation tubes at −20°C during the cruise and at −80°C after returning to the laboratory.

**Table 1:** Environmental parameters.

Sample	DO	COD	N-NO_3_	N-NH_4_	P-PO_4_	Temperature	Salinity	pH
C4_S	9.89	2.43	45.6	0.469	0.59	26.7	13.8	8.67
C5_S	6.04	0.63	39.4	1.066	0.34	24	23.2	8.18
D3_S	4.22	0.61	57.2	0.811	1.19	26	11.5	7.91
D4_S	5.69	0.66	38.8	0.998	0.34	24.5	20.4	8.15
D5_S	5.06	0.63	36	0.847	0.25	24.7	20.4	8.11
E3_S	4.27	0.8	50	0.472	1.02	24.5	18.1	7.91
E4_S	4.15	0.79	50.3	0.35	1.19	23.9	21.7	7.93
E5_S	7.97	2.03	63.9	1.011	0.51	25.7	19.6	8.44
F4_S	7.58	0.89	24.5	0.602	0.17	25.8	24	8.25
F5_S	7.97	0.84	10.2	0.416	0.17	27	26.3	8.18
C4_M	2.06	0.31	17.9	0.049	1.19	20.4	32.7	7.8
F5_M	2.32	0.29	4.6	0.214	0	26.5	31.3	7.94
F4_M	2.57	0.15	14.7	0.248	0.76	20.3	34	7.89
C5_M	2.12	0.3	26	-0.028	1.52	20.3	31.6	7.69
D3_M	1.7	0.31	20.9	0.19	1.61	20.3	33	7.72
D4_M	2.06	0.24	22.7	0.108	0.76	20.4	32.2	7.78
D5_M	2.28	0.31	29.5	0.211	1.02	21.1	30.1	7.81
E3_M	2.42	0.31	26	0.095	1.19	22.1	29.3	7.81
E4_M	2.34	0.3	26.3	0.168	0.93	21.3	30.5	7.8
E5_M	2.32	0.95	23.4	1.345	0.17	24.5	31.8	7.83
E5_B	2.6	0.31	15.8	0.176	0.76	20.3	34.2	7.84
D4_B	1.97	0.26	16.4	0.162	1.02	20	33.9	7.78
E4_B	2.21	0.4	10.8	0.464	1.19	20.3	34.2	7.81
F4_B	2.25	0.16	17.6	0.187	1.19	19.9	34.4	7.81
C5_B	2.26	0.37	13.9	-0.041	1.19	20.4	34.1	7.73
D3_B	1.68	0.37	19.2	0.214	1.19	20.01	33.2	7.73
F5_B	2.6	0.27	16.2	0.177	1.27	20.2	34.4	7.83
C4_B	2.05	0.34	15.6	0.293	1.36	20	34.1	7.78
D5_B	2.28	0.36	15.4	0.159	0.76	20.6	34.2	7.79
E3_B	2.54	0.26	20.6	0.165	1.1	20.4	33	7.82

Environmental parameters of the 30 water samples.

### Environmental parameters

Samples were pretreated according to specifications for marine monitoring (National Standards of People’s Republic of China, GB 17378.5, 2007) prior to chemical parameter analysis[11]. PO_4_^3–^ was measured using a QuAAtro continuous flow analyzer (SEAL Analytical, Hamburg, Germany). Temperature, dissolved oxygen (DO), and pH were measured using a YSI handheld meter. Chemical oxygen demand (COD) was measured using the alkaline potassium permanganate. NO_3_^−^ and NH_4_^+^ levels were measured using a spectrophotometer (752 UV/visible spectrophotometer, Shanghai-Hengping, China). Salinity was calculated by summarizing the concentrations of major ions.

### DNA extraction, PCR amplification, and sequencing

genomic DNA from each water sample was exacted using the FastPrep-24 rapid nucleic acid extraction kit (MP Biomedicals, USA) according to the manufacturer’s instructions. The concentration of purified DNA was measured using a protein nucleic acid detector (Bio-Rad, USA). Phylogenetically diagnostic sequences were amplified using the bacterial 16S rRNA universal primers 515F (5ʹ-GTGCCAGCMGCCGCGG-3ʹ) and 907R (5ʹ-CCGTCAATTCMTTTRAGTTT-3ʹ[12]). The PCR products were run on a 1.2% agarose gel in a TAE buffer and visualized by staining with SYBR Gold under UV light. Bands of the expected size were cut out and purified using the MiniBEST Agarose Gel DNA Extraction Kit Version 4.0 (TaKaRa, Japan) following the manufacturer’s instructions. They were then quantified using the NanoDrop ND-3000 (Thermo Scientific, USA). Purified DNA samples were sent for sequencing on a Miseq platform in the Majorbio (Majorbio, Beijing, China). QIIME software (http://qiime.org/scripts) was used to demultiplex the raw FASTQ file, and the paired reads were assembled with FLASH[13]. Then, sequences with insufficient quality (quality score <20) were discarded using QIIME. USEARCH was used to assess and filter the chimera of the sequences. The remaining sequences were clustered into operational taxonomic units (OTUs) with a complete linkage algorithm at a 97% sequence identity level(Padilla et al. 2015). Abundance-based coverage estimators, OTUs, the Chao1, Shannon, and Simpson parameters were calculated after rarefying all samples to the same sequencing depth of 28,153 sequences using QIIME software. Taxa with proportions <0.01% were grouped as “others. Non-metric multidimensional scaling (NMDS) was applied to reveal differences in community composition among different sites (using R 3.3.2 and Vegan package 2.4–1). Clustering based on the community structural or functional characteristics was performed using the R package Vegan. The heatmap.2 program within the gplots package was used to paint the heat map. With the Vegan package in the integrated suite of software facilities R[14], redundancy analysis (RDA) was used to examine the correlations between community variations and environmental parameters. Spearman correlations between abundant taxa and environmental factors were analyzed in R under using the ‘corrplot’ package. The relative abundances of the OTUs in each sample were used to construct matrices for visualizing interactions between OUTs in networks. A Spearman correlation coefficient R score and P-value were calculated pairwise between the OTUs (the OTUs with a relative abundance higher than 0.02%) using the Hmisc package (version 4.0–1) in R (version 3.3.2). These correlations were visualized using Cytoscape (version 3.4.0). Each node represented an OTU, and each edge represented correlations of the OTUs. To describe the network topology, a set of node/edge metrics were analyzed using the Network Analyzer plugin within Cytoscape[15].The modular structure analyses of the networks were also analyzed using the ClusterMaker in Cytoscape (version 3.4.0). The modularity of the networks was calculated[16]. Modularity values >0.4 suggested that the network was modular[17].

PICRUSt is a bioinformatics tool designed to address the functional potential in different sites using 16S ribosomal DNA sequences[18]. For this analysis, the closed-reference OTU picking protocol was performed using QIIME1.9.0[19]. Sequences are aligned using the Greengenes database (version. 13.5). The OTU table was created after rarefying samples to 12,239 sequences, and then the gene copies were normalized. Finally, further analysis was conducted using PICRUSt. These results were used to choose the functional analysis for COGs and KEGG ortholots. Here, the KEGG ortholots (KOs) were used. The relative abundance of functional categories was generated using the OTU table of assigned taxa and their relative distribution in different samples[20]. In the KEGG database, functions were grouped into three level subgroups based on different KEGG (i.e. metabolism, cellular processes, and environmental processing).

All the sequences in this study have been submitted to the NCBI-SRA public database (http://www.ncbi.nlm.nih.gov/sra/SRP149966) under the ID: SRP149966 (all the thirty samples of the Changjiang Estuary).

## Results

### Statistical information of the environmental characteristics

**Fig 2:**
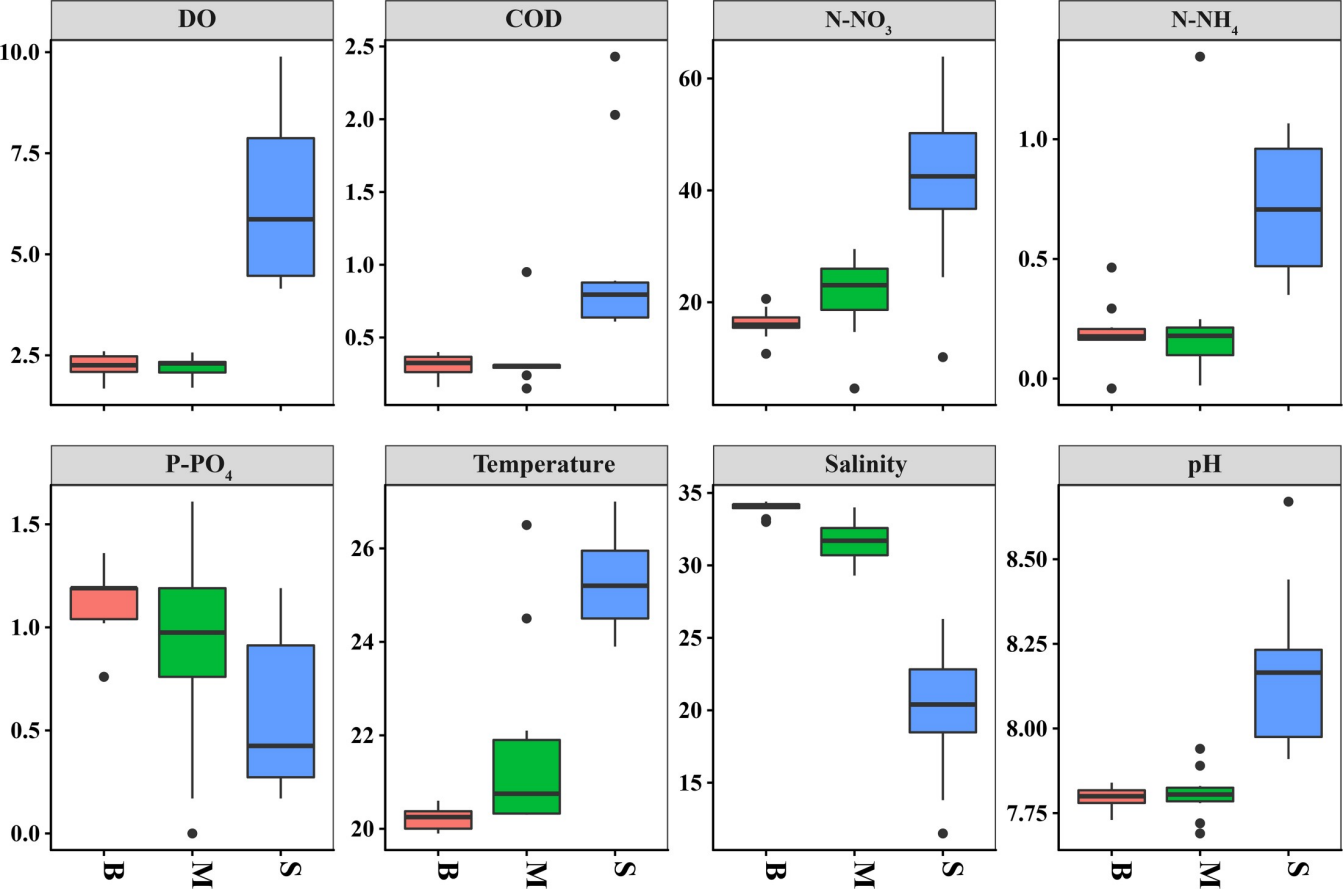
The environmental factors of three water layers. Box plots for environmental factors in different water layers.

The samples from the three sea water layers showed strong variations in chemical oxygen demand (COD), dissolved oxygen (DO), ammonia nitrogen (N-NH_4_), nitrate nitrogen (N-NO_3_), orthophosphate (P-PO_4_), pH, and salinity (Table 1). In this study, it was found that COD, DO, N-NH_4,_ and N-NO_3_ were higher in the surface layer, while P- PO_4_ and pH were higher in the bottom layer (Fig 2). The DO content of the middle layer and bottom layer were extremely low (< 2.5 mg L^-1^), indicative of severe hypoxia. However, the temperature showed no statistical difference among three water layers. The value of each environmental variation is shown in Table 1.

### Richness and diversity analyses based on operational taxonomic units

**Fig 3:**
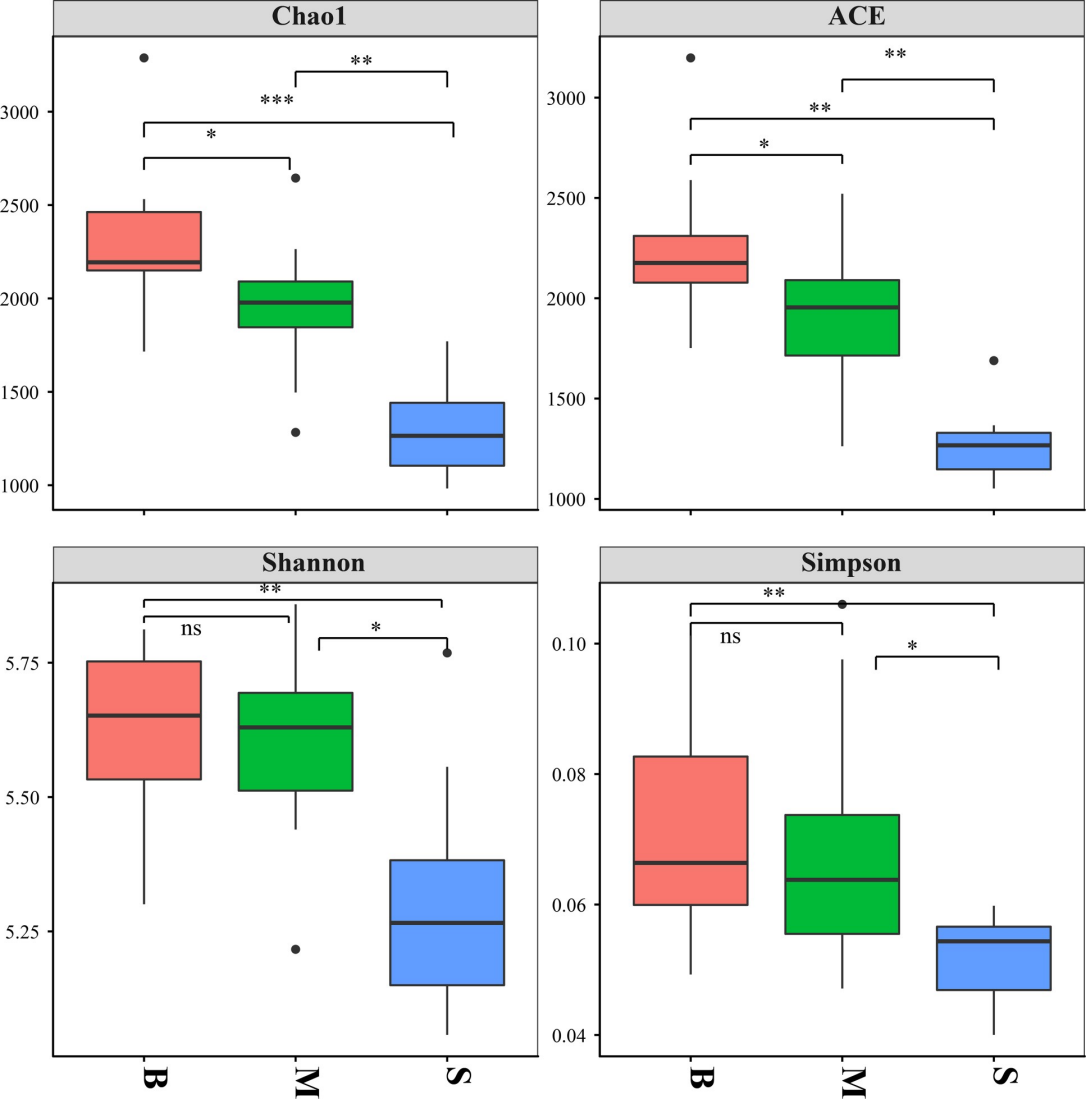
The alpha-diversity of bacteria. Box plots for alpha-diversity of the bacterial communities in different water layers.

A total of 1,094,062 raw reads were obtained from all of the 30 samples. 1,044,722 sequences were retained for further analysis (S1 Table). To compare the diversities and richness of bacterial communities in the different water layers, Chao1, ACE, Simpson, and the Shannon-Weaver diversity index were calculated (Fig 3). The richness, estimated by the number of ACE and Chao1, increased from the surface layer to the bottom layer. The diversity, indicated by the Shannon diversity indices, also showed the same trend. All these showed that bacterial abundance and diversity in the hypoxic zone was higher than that in the non-hypoxic zone.

### Similarity of bacterial communities in the different water layers

**Fig 4:**
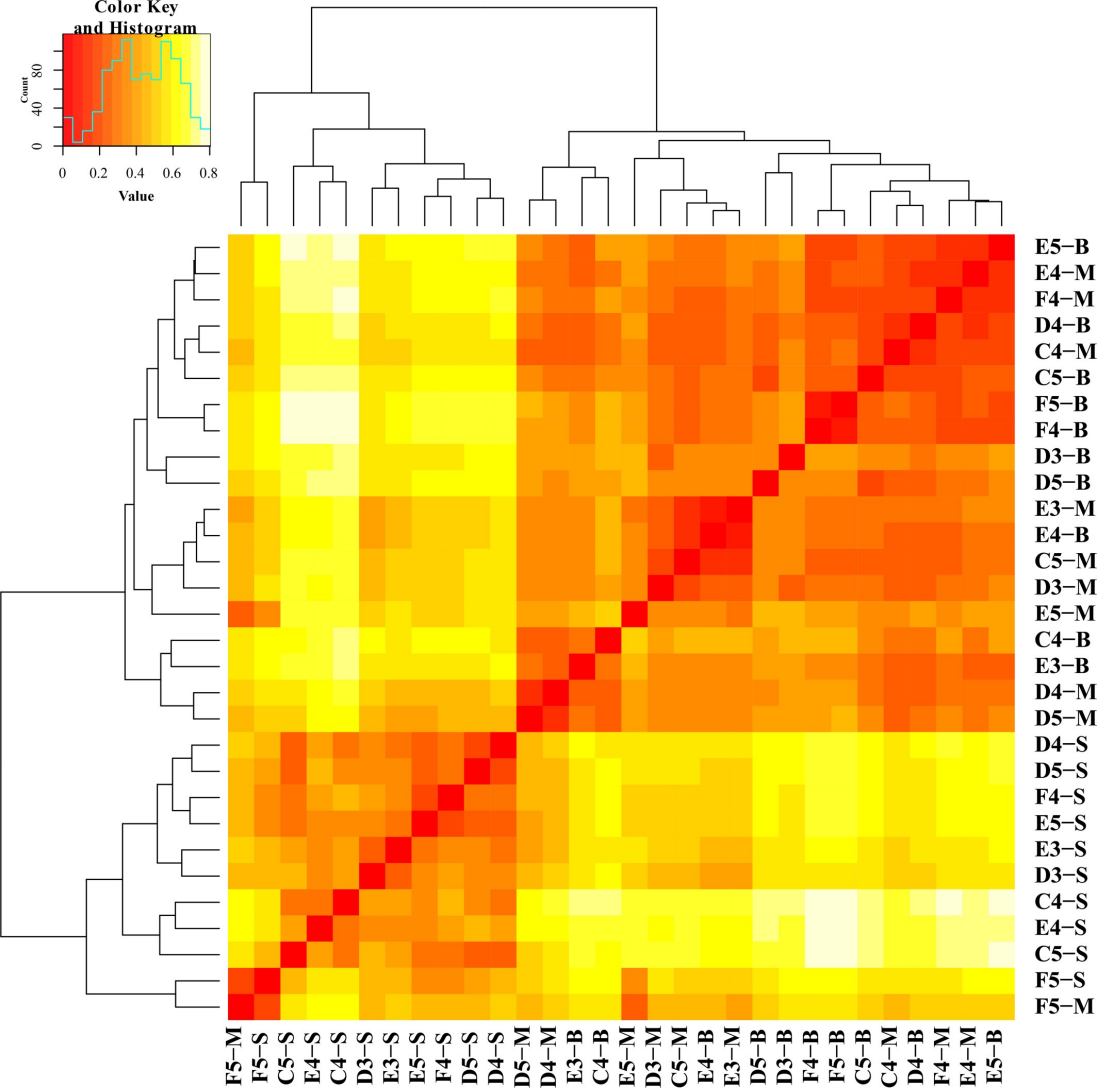
The taxonomic differences of microbial communities among different sites. Heatmap representing the differences among samples based on the Bray-Curtis distance.

**Fig 5:**
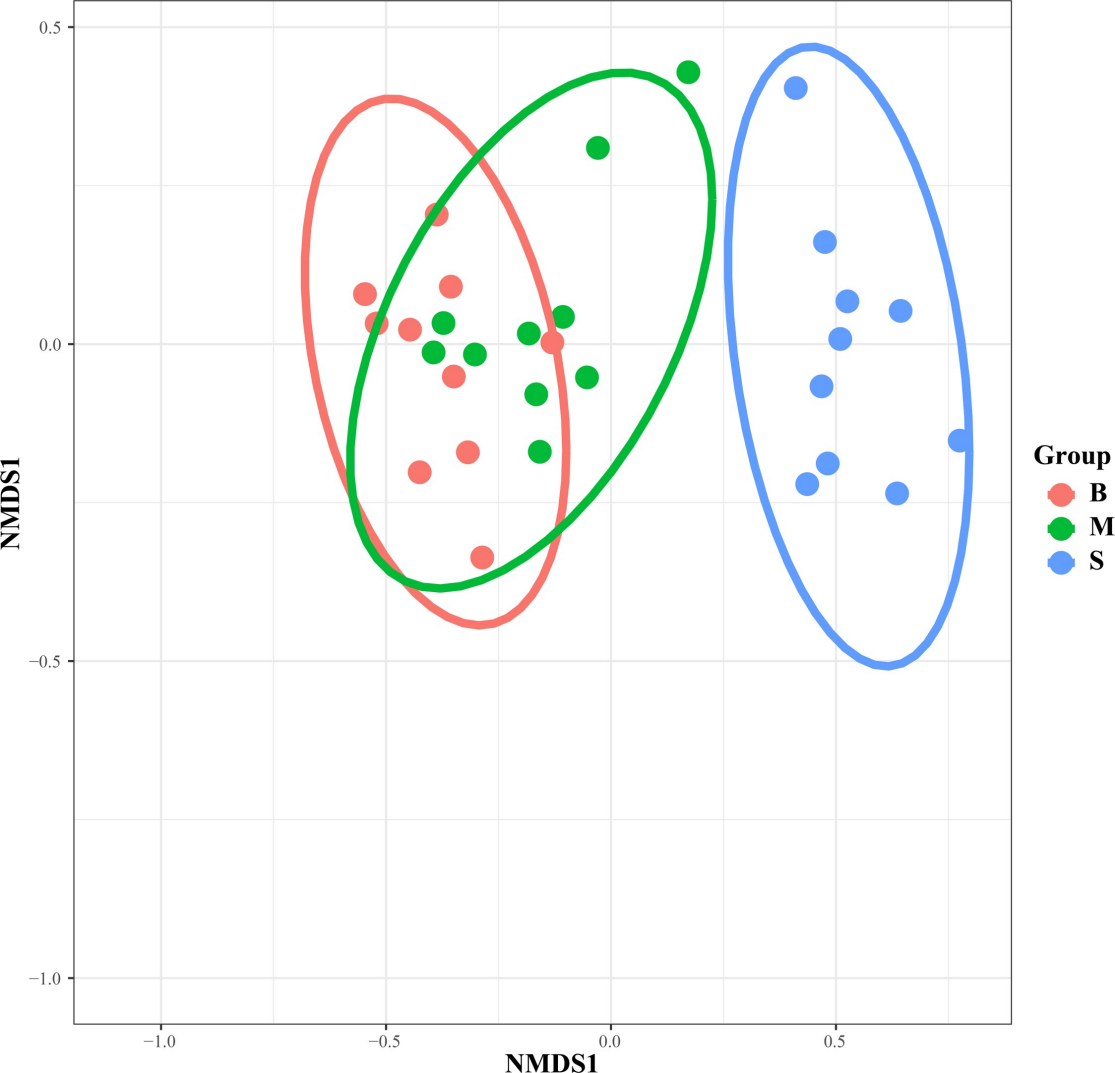
The taxonomic differences of microbial communities among different sites. Non-metric multidimensional scaling analysis (NMDS) of the dissimilarities among microbial communities using the Bray-Curtis distances.

To examine the clustering patterns of samples from different water layers, a heatmap (Fig 4) and NMDS (Fig 5) were generated based on the Bray-Curtis distance. Samples from the middle and bottom layers (hypoxic zones) were clustered together and separated from samples from the surface layer (non-hypoxic zones).

### Bacterial community composition and structure in different water layers

**Fig 6:**
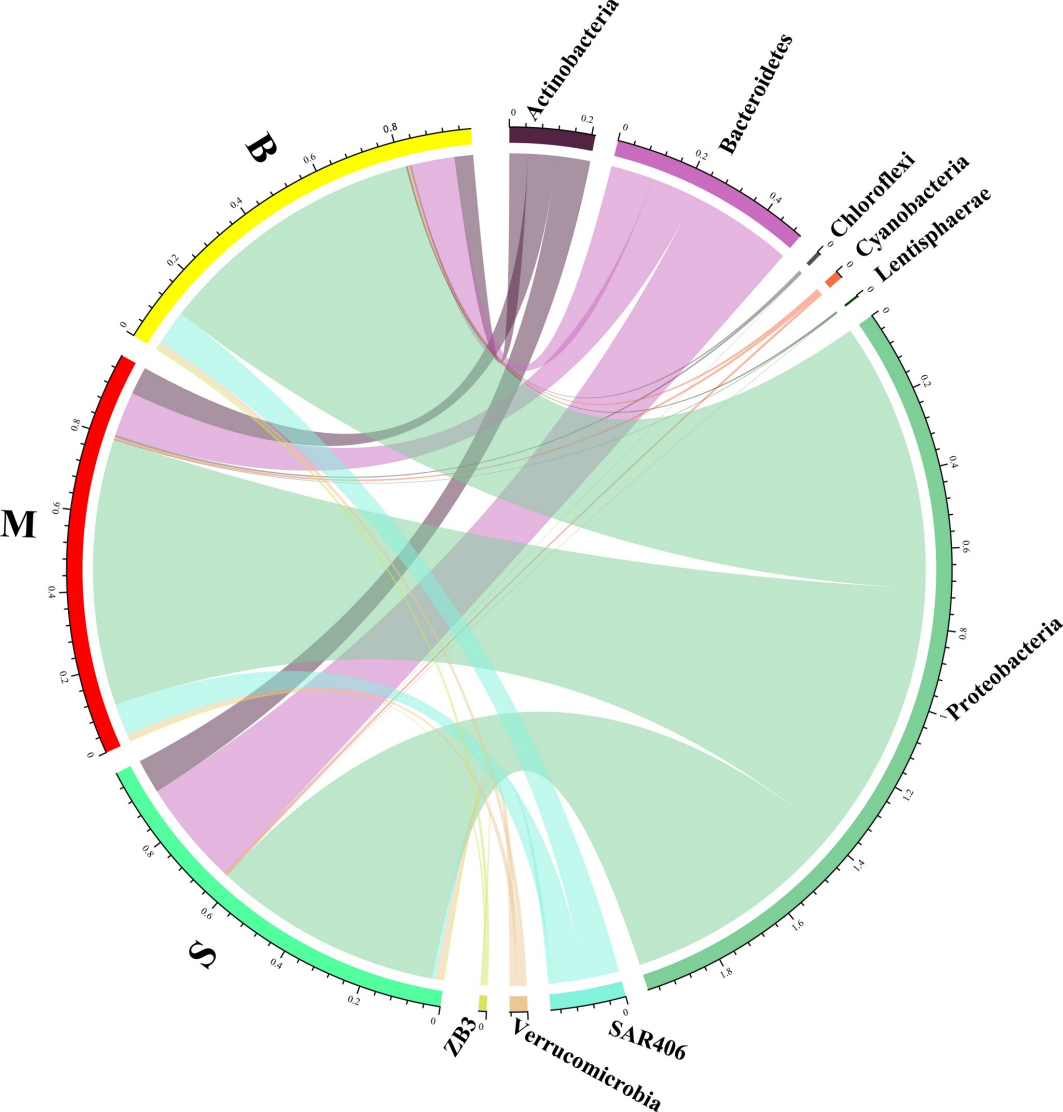
Microbial composition at phylum level. The circus plot showing the community structure of the 30 water samples at the phylum level.

**Fig 7:**
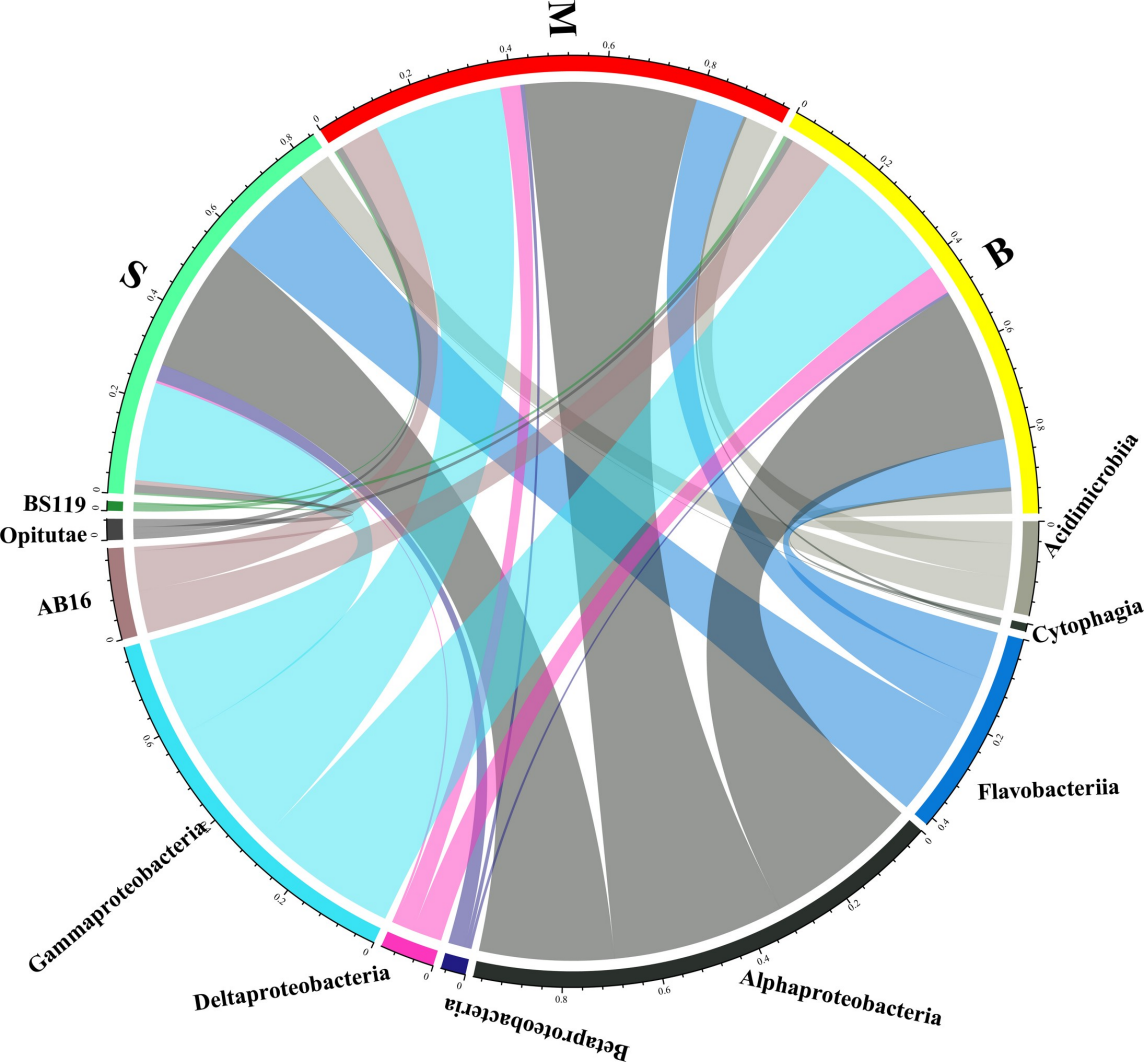
Microbial composition at class level. The circus plot showing the community structure of the 30 water samples at the class level.

QIIME software was used to identify sequences. There were a total of 37 phyla, and 89 classes in all samples. The composition of the bacterial communities in different layers were compared at the phylum level (Fig 6) and the class level (Fig 7). Proteobacteria was the most abundant phylum in all layers, followed by Bacteroidetes, Actinobacteria and SAR406. It was also shown that Bacteroidetes was the predominant phylum in the surface layer, while SAR406 primarily dominated in the middle and bottom layers. At the class level, Alphaproteobacteria was abundant in all the layers. Deltaproteobacteria and Gammaproteobacteria had higher relative abundance in the middle and bottom layers (hypoxic areas) while Flavobacteria and Beltaproteobacteria were more abundant in the surface layer (non-hypoxic areas). Further analysis at the order and family levels showed that the abundance of the bacterial community had significant differences among the water layers (S2 Table).

### Influence of environmental factors to bacterial community structure

**Fig 8:**
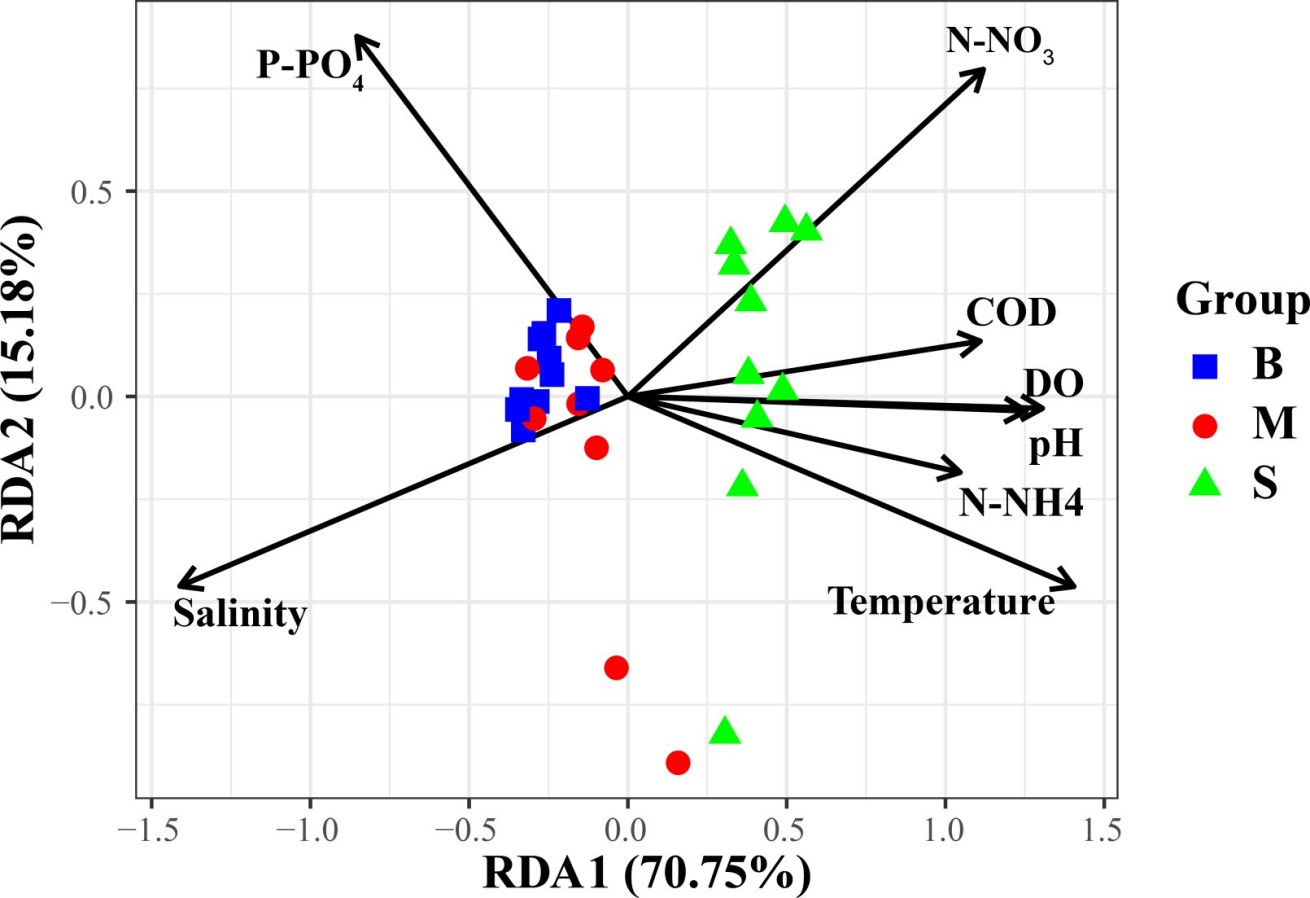
Relationship of environmental factors and bacterial communities in the Changjiang Estuary. Redundancy analysis (RDA) shows the relationships between environmental variables and the bacterial communities in the Changjiang Estuary.

**Fig 9:**
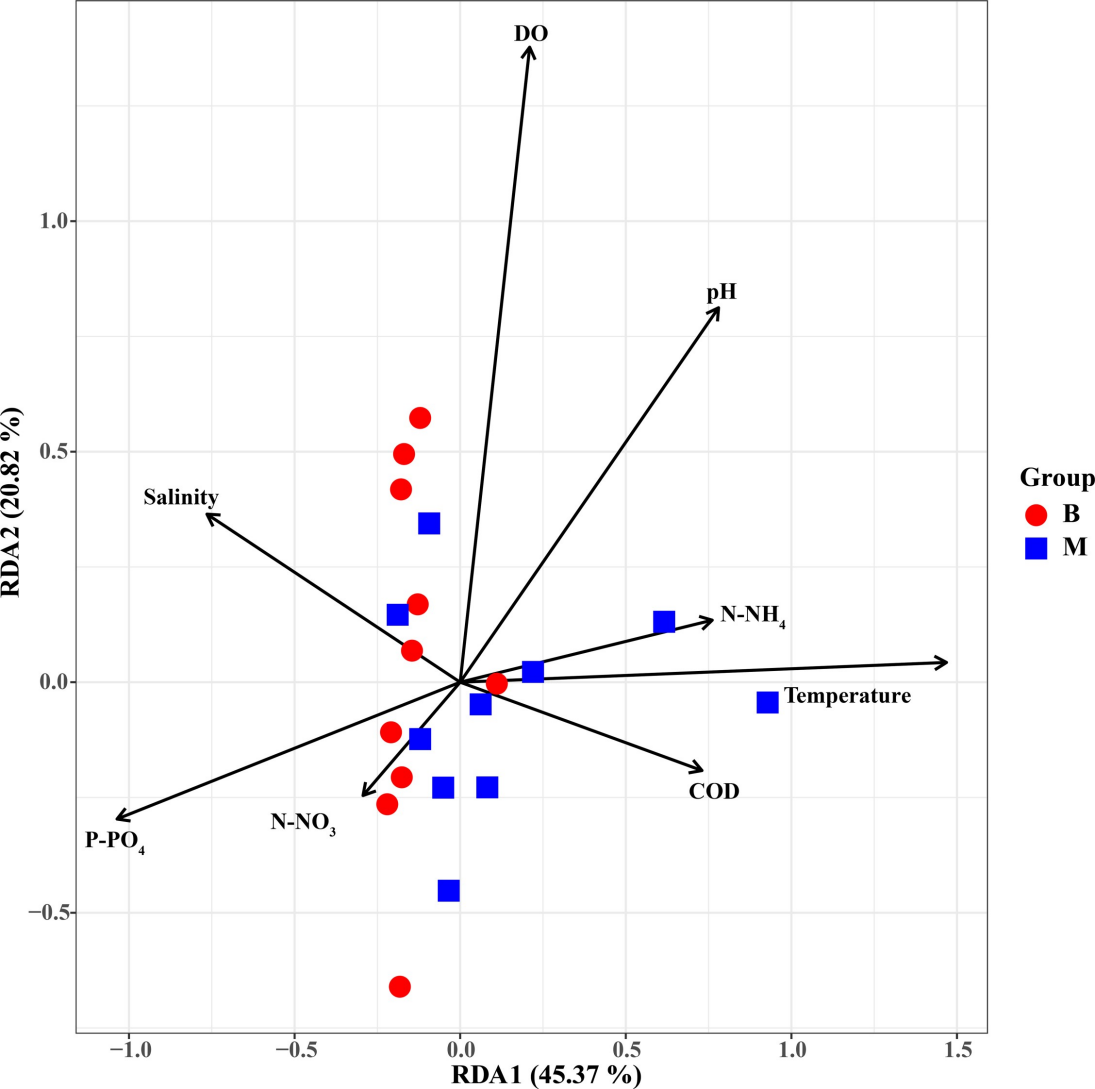
Relationship of environmental factors and bacterial communities in the hypoxic zone. Redundancy analysis (RDA) shows the relationships between environmental variables and the bacterial communities in the hypoxic zone.

**Fig 10:**
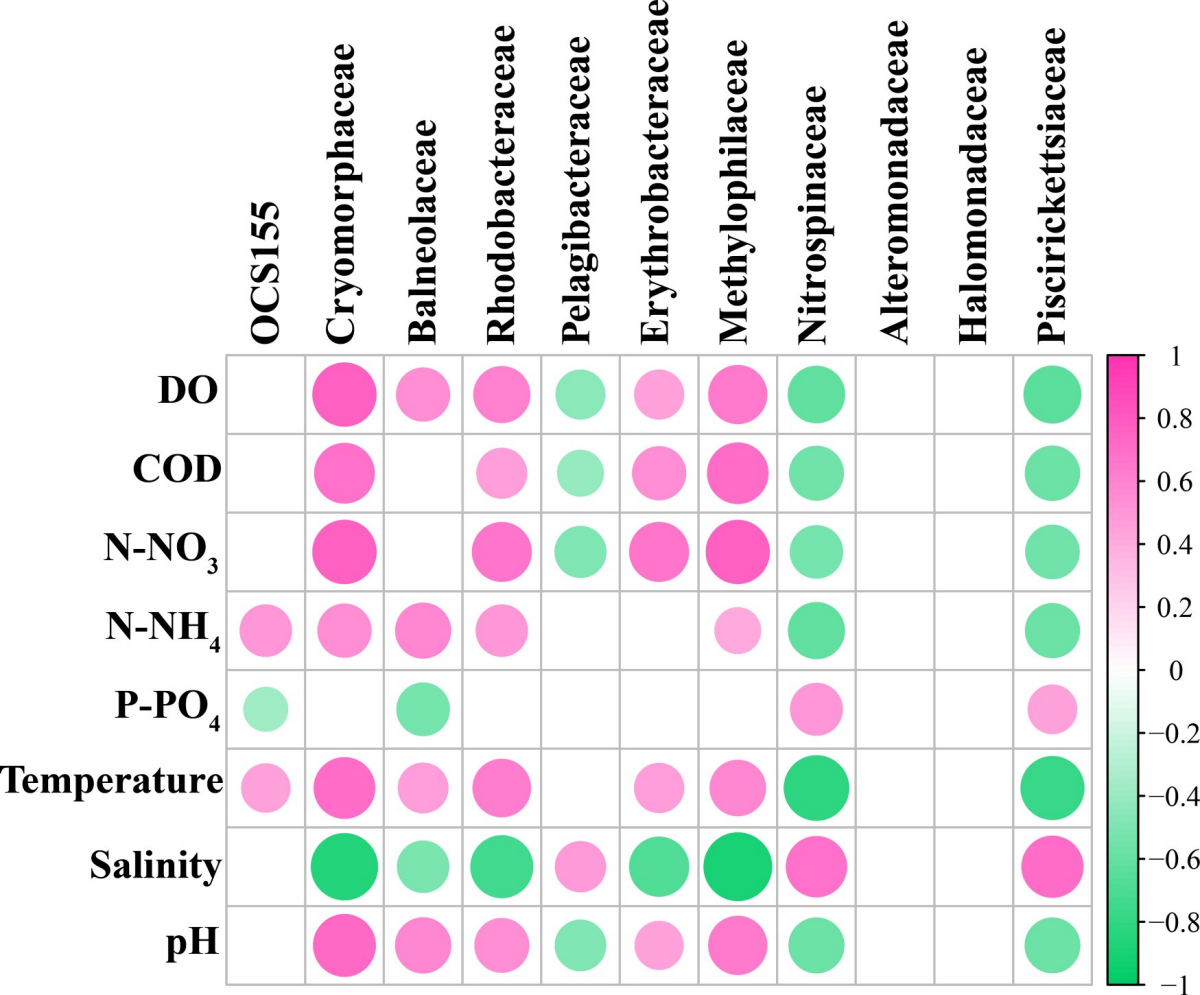
Relationships of environmental factors and dominant order. Correlations between the dominant order and water environmental variables.

The results of this study indicated that environmental factors are important determinants of microbial communities in the water samples. To explore how environmental parameters influenced the bacterial community in the hypoxic zones, RDA was performed. Results indicated that P-PO_4_, N-NO_3_, and salinity showed relationships with taxonomic composition in the Changjiang Estuary (Fig 8). The first two axes explained 70.75% of the taxonomic information. In the hypoxic zone, it was found that DO, P-PO_4,_ temperature, and pH had significant influence on the bacterial community (Fig 9). DO accounted for most of the bacterioplankton variation in the RDA targeting only hypoxic zones, remarkably different from that targeting all samples in the Changjiang Estuary. Hence, DO was a major environmental factor that influenced structure of the microbial community (P = 0.001) in the hypoxic area. The envfit showed that P-PO_4_ and DO had significant inflence on the bacterial communities (P = 0.001) (Tables 2 and 3).Correlation analysis showed that Flavobacteriales and Methylophilales were positively related to DO while Rhodobacterales and Thiohalorhabdales showed a negative correlation (Fig 10). Rhodobacterales and Methylophilales seemed to be negatively related to the salinity while Rhodospirillales and Thiohalorhabdales had a positive correlation with it.

**Table 2:** The result of redundancy analysis in the Changjiang Estuary.

	RDA1	RDA2	r^2^	P
DO	0.99750	-0.7066	00.6897	0.003**
COD	0.99725	0.07416	0.4984	0.003**
N-NO_3_	0.82170	0.56991	0.7294	0.001***
P-PO_4_	0.97561	-0.21952	0.4635	0.001***
N-NH_4_	-0.69514	0.71888	0.6369	0.001***
Temperature	0.93627	-0.35129	0.9090	0.001***
Salinity	-0.96135	-0.27533	0.8652	0.001***
pH	0.99706	-0.07656	0.6364	0.001***

Redundancy analysis of environmental factors and bacterial community structure in the Changjiang Estuary. The symbol ** and *** represents the significance (P<0.05).

**Table 3:** The result of redundancy analysis in the hypoxic area.

	RDA1	RDA2	r^2^	P
DO	0.17639	0.98432	0.7247	0.001***
COD	0.97509	-0.22182	0.2326	0.117
N-NO_3_	-0.79737	-0.6035	0.0585	0.585
P-PO_4_	0.98619	0.16561	0.2466	0.105
N-NH_4_	-0.96638	-0.25713	0.4781	0.012*
Temperature	0.99938	0.03534	0.8913	0.001***
Salinity	-0.92147	0.38845	0.2886	0.06
pH	0.72986	0.6836	0.501	0.002**

Redundancy analysis of environmental factors and bacterial community structure in the hypoxic area of the Changjiang Estuary. The symbol *, ** and **** represents the significance (P<0.05).

### Bacteria co-occurrence

To investigate the relationship between different phyla, co-occurrence networks of the three water layers were built for the Changjiang Estuary (S1,S2 and S3 Figs). Overall, the surface layer microbial network contained 284 nodes (i.e. OTUs) with 7,603 edges (significant correlations), while the middle layer microbial network contained 423 nodes with 12525 edges. The bottom layer microbial network contained 463 nodes with 12,583 nodes. Moreover, the middle and bottom layers (hypoxic areas) microbial assemblages had a more correlated and complex bacterial network topology than the surface layer (non-hypoxic areas). Compared the topological parameters of these three networks (S3 Table), the middle and bottom layer microbial networks exhibited a greater number of edges, number of communities, average degree, average clustering coefficient, and average path length (S3 Table).

### Predicted metabolic analysis

**Fig 11:**
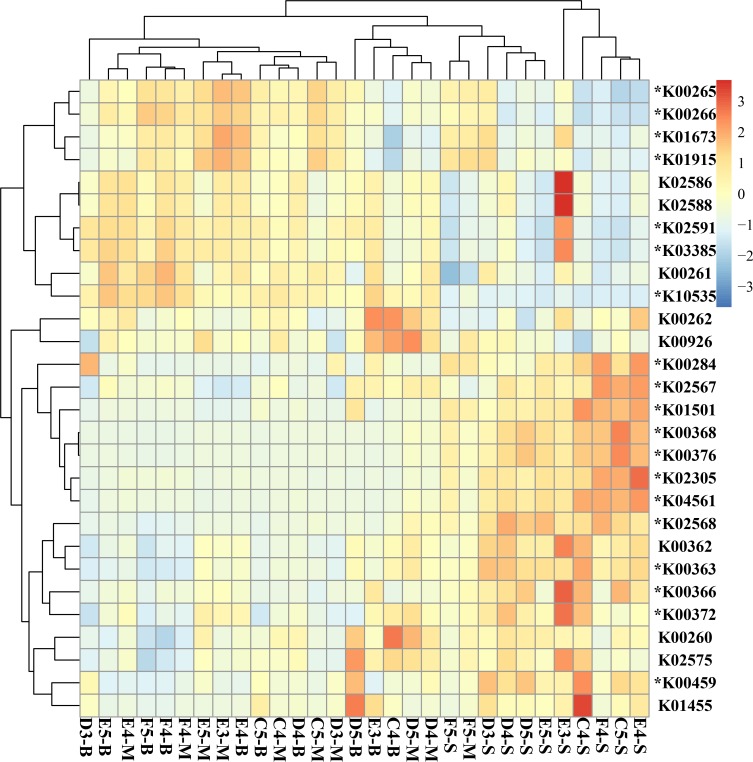
The KEGG orthology groups involved in the nitrogen metabolism pathways. Heatmap showing the differences among the three water layers based on the KEGG orthology groups involved in the nitrogen metabolism pathways. An asterisk indicates there was a significant difference among the surface layer, the middle layer and the bottom layer.

PICRUSt was used to investigate the community functions. The functional categories were classified as level-2 and level-3. A majority of the KOs at level-2 involved metabolism, cellular processes, organismal systems, genetic information processing, environmental information processing and unclassified processes. These genes were significantly different between the hypoxic and non-hypoxic areas. In the middle and bottom layers (hypoxic areas), the bacterial community had more relative genes associated with metabolism, cellular processes, organismal system and genetic information processing while environmental information processing was abundant in the non-hypoxic areas (ANOVA test, all P < 0.05, S4 Table).To gain more insight into nitrogen metabolism, the functional genes that are involved in nitrogen metabolism were also analyzed. Results showed that a majority of the genes were significantly different among the surface layer, the middle layer, and the bottom layer (Fig 11). The KOs predicted to be enriched in both the middle and bottom layers (hypoxic areas) included K02586 (*nifD*), K02588 (*nifH*), K03385 (*nrfA*), K10535 (*hao*) while K00368 (*nirK*), K00376 (*nosZ*), K02305 (*norC*), K00372 (*nasA*), K00366 (*nirA*), K04561 (*norB*), K02568 (*napB*) and K00363 (*nirD*) were abundant in the surface layer (non-hypoxia). For further analysis, the oxidation state was investigated (S4 Fig). The result showed that the bacterial community in the surface layer (non-hypoxic areas) had more genes related to anaerobic ammonium oxidation (anammox) and denitrification. In addition, the bacterial community in the hypoxic areas had more genes involved in nitrification and nitrogen fixation (S5 Fig).

## Discussion

### Comparison of dominant taxa in the different water layers

In this study, it was found that the richness and diversity of bacteria increased from the surface layer to the bottom layer. This is consistent with the results of a previous study conducted in the Mediterranean Sea [21]. Sunagawa *et al* also found the global relevance of this pattern [22]. The increase in taxonomic richness and diversity might reflect diversified species adapted to a wider range of niches, such as particle-associated micro- environments in the middle and bottom layers [23]. In this study, we found that Alphaproteobacteria, Gammaproteobacteria, and Bacteroidetes were the dominant groups, which is similar to other bacterial communities in marine waters [24–27]. Alphaproteobacteria had the highest abundance and dominated in all the water layers. Alphaproteobacteria is generally ubiquitous in marine waters [28, 29]. A previous study reported that Alphaproteobacteria was frequently distributed in oligotrophic natural ecosystems [30]. Rhodobacterales, Rickettsiales, and Sphigomonadales affiliated with Alphaproteobacteria were dominant in the all the water layers. Sphingomonadales has been reported to have metabolic capabilities [31] and plays a key role in the degradation of aromatic compounds [32]. Rickettsiales was the most abundant order in all the water layers. Previous studies reported that Rickettsiales were abundant in ocean water [33, 34] and were negatively correlated to DO (Fig 10). Rhodobacteraceae (Class Alphaproteobacteria) was highly abundant in the surface layer, which is in agreement with the result of a previous study that reported they are rapid surface colonizers and photosynthetic bacteria [35]. Oceanospirillales was significantly distributed in the bottom layer and only positively correlated with salinity (Fig 10). During summer, marine water enters the estuary bottom [36], Oceanospirillales are of ocean origin and may come from the surface water of the East China Sea. In this study, it was also found that Methylophilales was the significant order in the surface water layer. Correspondingly, Methylophilales has been reported to be dominant in oxic surface water and related to methylotropic metabolism [37]. Desulfobacterales (Order Deltaproteobacteria) was primarily distributed in the bottom layer, which was consistent with a previous study that found that Desulfobacterales are a diverse group of anaerobic bacteria, that use sulphate as terminal electron acceptor in the oxidation of H2 and organic compounds [38]. They are known to play an important role in C and S cycling and in the degradation of organic contaminants [39, 40]. Rickettsiales appeared to be more abundant in the middle and lower water columns [41]. Correspondingly, in this study, Rickettsiales was significantly abundant in the middle and bottom layers. Previous studies reported that SAR406 was a common clade in open ocean waters [42] and were abundant in the bottom water column [41]. The results of this study that showed that SAR406 was primarily distributed in the middle and bottom layers is consistent with the results of the previous study.

### The influence of environmental factors on the bacterial community

Hypoxia has been reported in estuarine environments [43]and it has an impact on the bacterial community in many water systems [3–5, 44]. In this study, it should be noted that DO was remarkably lower in the hypoxic area and had the greatest impact on bacterial composition. These results agree with a previous study that showed DO accounted for most of the bacterioplankton variation in the hypoxic area of the Pearl Estuary [44]. However, Liu *et al* studied the bacterial community in the Changjiang Estuary hypoxic area and found that DO had no significant correlation with the bacterial community [8]. This may have been caused by the different sampling sites and different months. In the oxygenated surface waters (10 m), the microbial community structure was dominated by aerobic heterotrophic bacteria that have been commonly observed in pelagic marine environments [45] and the most prevalent genus was a-proteobacteria affiliated with the Rhodobacterales. This result is consistent with results found in this study that Rhodobacterales was notably abundant in the surface layer (non-hypoxic area) and was had a positive correlation with DO (Fig 10). Methylophilales was the most dominant order in the surface water layer and had a positive correlation with DO (Fig 10). Correspondingly, Methylophilales has been reported to be dominant in oxic surface waters and it is related to methylotropic metabolism [37]. One study clearly demonstrated the structuring effect of dissolved oxygen, because some bacterial communities have an advantage in the hypoxic zone, likely due to their use of using alternative electron acceptors (e.g. denitrification) [46]. Bacterial communities are always closely related to their environments, and factors such as salinity, temperature and nutrients can be used as an indicator of marine ecosystem status [47]. In the non-hypoxic area, it was observed that P-PO_4_ was the most important factor that affected affecting the bacterioplankton community. The Changjiang Estuary and its adjacent areas are a complex system that has water that mixes with the TWC. The TWC originates from the Taiwan Strait, that extends along the coast of the Fujian and Zhejiang Provinces to the north and meets with Changjiang Estuary. Yang et al. found that the sources of phosphate in the Changjiang Estuary included the Changjiang River, the TWC, the cyclone-type eddy and the 32°N upwelling [48]. These may result in the high phosphate concentration in the Changjiang Estuary, the variation in which correlated significantly with the bacterial community. In this study, the RDA results indicated that temperature, salinity, and pH also affected the bacterial community in the hypoxic area. From a previous study, it is known that temperature is the most important factor that affects bacterial communities [49]. Wells *et al*. (2011) also reported that temperature might be a reflection of seasonal periodicity in bacterial communities [50]. It was observed in this study that temperature had a significant positive correlation with Flavobacteriales and Rhodobacterales, but a negative correlation with Rhodospirillales and Thiohalorhabdales (Fig 8). pH has been reported to have a significant impact on bacterial abundance and diversity [51, 52]. Most microorganisms are usually within one pH unit of neutral, and a remarkable deviation in the environmental pH would impose stress on single-celled organisms [53]. Salinity also had a significant influence on the microbial community in this study. It had positive correlation with Nitrosopinaceae and Piscirickettsiaceae, but was negatively correlated with Cryomorphaceae, Methylophilaccac and Rhodobacteraceae (Fig 10). This is consistent with previous studies that have found that salinity and temperature are key environmental factors that shape microbial community structure in different environments and explain a significant portion of global microbial community distribution patterns [22, 54].

### Co-occurrence patterns of the three water layers

Network analysis has been used to investigate microbial co-occurrence in many complex environments. Co-occurrence patterns are important to understand microbial community structures, and they offer new insights into potential interaction networks, which reveal niche spaces shared by community members. The network topology (the nodes distribution and interaction) affects the stability of systems [55, 56]. In macroecology, communities with tightly connected species are more susceptible to disturbance [57, 58]. In this study, the high heterogenous habitats in the non-hypoxic area and the high homogeneous habitats in hypoxic area might result in the different topological structures of these three networks. A highly connected microbial network in the middle and bottom layers suggests that microbial communities that are close to the middle and bottom layers are more vulnerable and sensitive to various disturbances. In the surface layer microbial network, 33% of the correlations were negative, with 42% and 40% in the middle layer and bottom layer, respectively (S2 Table). This indicates that microorganisms in the middle and bottom layers have more competing relationships than microorganisms in the surface layer. The limited resources in the middle and bottom layers may lead to this. The nutrients (N-NH_4_, N-NO_3,_ and DO) were significantly lower in the middle and bottom layers. The bacterial assemblages in these three water layers exhibited a modular structure (S2 Table, a modularity value > 0.4 suggests that the network is modular) [17]. Modularity is a characteristic of large complex systems[17, 59]. In a biotic network, highly interconnected species are grouped into a module, within which species interactions are more frequent and intensive than with the rest of the community[59]. The higher modularity might be due to more pronounced niche differentiations[60]. It was found that the modularity value of the middle and bottom layer microbial networks were notably higher than the surface layer microbial network (S2 Table). From the network (S1, S2 and S3 Figs), it can also be observed that the number of communities in the middle and bottom layers were more than the surface layer, indicating that middle and bottom layer environments offered more niches for organisms. This is consistent with the result of our previous study that found that the bacterial richness and diversity of the middle and bottom layers were significantly higher than the surface layer. This result reveals that the hypoxic area may provide a more suitable environment for the bacterial communities.

### Nitrogen metabolism

Nitrogen plays key role in biogeochemical processes[61] and involves various microbe-derived enzymes[62]. To enhance our understanding of denitrification in an hypoxic area system, it is necessary to know the process of nitrogen metabolism and how bacteria respond to environmental factors. Nitrogen metabolism includes seven pathway types: nitrogen fixation, nitrate transport system, nitrification, dentification, dissimilatory nitrate reduction, assimilatory nitrate reduction, and complete nitrification. Assimilatory nitrate reduction and dissimilatory nitrate reduction all begin with nitrate to nitrite then to ammonium. The Dissimilatory nitrate reduction can retain nitrogen in the system in a bioavailable form (NH_4+_) for further biological processes[63, 64], while assimilatory nitrate reduction can be readily used by cells for synthesis of amino acids and nucleotides[65, 66]. Denitrification is the process of reductive respiration and uses nitrate or nitrite to nitric oxide or nitrous oxide and then to nitrogen. The Denitrification is encoded by genes (*norB*, *nosZ*, *napA* and *norC*). The *nosZ* gene is primarily unique to denitrifying bacteria and some non-denitrifier species. It also plays a role in the process of reducing nitrous oxide[67, 68]. Members of Comamonadaceae have been shown to be involved in denitrification[3, 69]. This is line with the results of this study that found that Comamonadaceae was abundant in the surface layer. In this study, it was found that the genes related to denitrification process in the surface layer (non-hypoxic area) were more numerous than in the hypoxic area. Hence, we speculated that the abundance of denitrifying bacteria was higher in the surface layer (non-hypoxic area). Another hypothesis would be that there were many aerobic denitrifiers in the surface layer. Previous studies have shown that aerobic denitrification can occur in many marine systems[70, 71]. However, further studies should be performed to investigate the aerobic denitrifiers and denitrifying bacteria in the waters of the Changjiang Estuary. Nitrification is one of the important processes in nitrogen metabolism that converts the ammonia to hydroxylamine, nitrite, and then to nitrate. The main genes include *hao*, *pmoa-amoA*, *pmob-amoB* and *pmoc-amoC*. Nitrogen fixation is encoded by *nifD*, *nifK* and *nifH*. The *nifD* gene plays a key role in the biological conversion of atmospheric nitrogen to ammonia or nitrogen fixation[72]. Anammox is also a significant process in the nitrogen metabolism. The bacteria that perform the anammox process belong to the bacterial phylum Planctomycetes[73]. In this study, the abundance of Planctomycetes was relatively low and were primarily distributed in the hypoxic area. However, it was found that the gene involved in anammox was *nirK* and had the highest abundance in the surface layer. It was speculated that there were many aerobic ammonia-oxidizing bacteria in the surface water of the Changjiang Estuary. However, more studies that investigate this bacteria need to be conducted in the future.

## Conclusion

Bacterial community structure was significantly different between hypoxic and non-hypoxic areas. Meanwhile, the bacterial richness and diversity were decreased with water depth. We detected 37 phylum, including Proteobacteria, Acidobacteria, Actinobacteria, Bacteroidetes and unclassified, as well as others with low abundance. Alphaproteobacteria and Gammaproteobacteria were the predominant classes in all water samples. In the hypoxic area, SAR406 and Deltaproteobacteria were the dominant groups, while Flavobacteriia, Acidimicrobiia, and Beltaproteobacteria were dominant groups in the non-hypoxic area. RDA results showed that DO had the greatest impact on the bacterial community in the hypoxic zone. These findings expand our current understanding on bacterial structure and function in hypoxic and non-hypoxic areas of the Changjiang Estuary.

## Supporting information

S1 Table Bacterial diversity indices. The alpha-diversity of bacterial communities.
(PDF)

S2 Table The Relative abundance of the major bacteria. Relative abundance (the mean values and standard deviations, std) of the major bacteria in the different water layers. An asterisk indicates a significant difference between the surface layer, the middle layer, and the bottom layer (ANOVA-test, P <0.05).
(PDF)

S3 Table The parameters of networks. Simple parameters of different water layers in the Changjiang Estuary network.
(PDF)

S4 Table Relative abundance of the functional genes. Relative abundance (the mean values and standard deviations, std) of the functional genes in the different water layers. An asterisk indicates a significant difference between the surface layer, the middle layer and the bottom layer (ANOVA test, P <0.05).
(PDF)

S1 Fig The co-occurrence network of bacterial communities in the surface layer. The co-occurrence network of bacterial communities in the surface layer. Edges represent correlation relationships. The nodes are sized by OTU betweenness and colored by phylum.
(PDF)

S2 Fig The co-occurrence network of bacterial communities in the middle layer. **The co-occurrence network of bacterial communities in the middle layer.** The edges represent correlation relationships. The nodes are sized by OTU betweenness and colored by phylum.
(PDF)

S3 Fig The co-occurrence network of bacterial communities in the bottom layer. The co-occurrence network of bacterial communities in the bottom layer. The edges represent correlation relationships. The nodes are sized by OTU betweenness and colored by phylum.
(PDF)

S4 Fig The oxidation state of nitrogen. A map of the nitrogen cycle constructed using the KEGG nitrogen metabolism. It includes dissimilatory nitrate reduction, assimilatory nitrate reduction, denitrification, nitrogen fixation, nitrification and anammox. There are a variety of genes that encode enzymes that catalyze the important transformation reactions of various nitrogen forms ranging from oxidation states of +5 in nitrate to −3 in ammonium.
(PDF)

S5 Fig Relative abundance of the major categories of functional genes. Relative abundance of the major categories of functional genes that encode the enzymes that catalyze nitrogen cycling pathways (dissimilatory nitrate reduction, assimilatory nitrate reduction, denitrification, nitrification, nitrogen fixation, and anammox) based on the KEGG database.
(PDF)
