# Supplementary material for: Comparison of bacterial community structure and potential functions in hypoxic and non-hypoxic zones of the Changjiang Estuary
Source: PLoS One. 2019 Jun 6;14(6):e0217431. doi: 10.1371/journal.pone.0217431 (PMC6553723; doi:10.1371/journal.pone.0217431)
Supplement: S1 Table — The alpha-diversity of bacterial communities. (PDF) [file pone.0217431.s001.pdf]

S1 Table

Sample	Raw reads	Clean reads	chao1	shannon	simpson	otus	ace
C4_S	34964	33728	1241.264	5.328391	0.959971	499	1285.539
C5_S	44347	43186	1083.372	5.079369	0.946833	433	1114.699
D3_S	34561	33021	1466.2	5.556246	0.943695	675	1366.511
D4_S	39219	37632	1077.015	5.333416	0.953676	539	1081.216
D5_S	36761	35504	982.9322	5.057565	0.943296	468	1052.206
E3_S	32736	30471	1770.012	5.768274	0.957823	750	1689.079
E4_S	32007	30547	1287.781	5.398698	0.95137	587	1245.156
E5_S	34484	32721	1377.457	5.198936	0.944438	510	1286.526
F4_S	37199	34760	1167.525	5.133564	0.940195	518	1248.49
F5_S	45139	43913	1462.528	5.203226	0.941629	553	1343.538
C4_M	31166	28153	2096.161	5.67949	0.937943	957	2114.053
F5_M	44672	42976	1282.884	5.583039	0.944964	611	1262.423
F4_M	39842	36180	1936.1	5.698709	0.929829	917	1945.58
C5_M	32119	29698	2019.5	5.216652	0.893981	847	1963.244
D3_M	33049	28804	2644.446	5.676153	0.925091	1056	2520.519
D4_M	36918	33170	1872.348	5.858698	0.952864	836	1725.805
D5_M	35469	32225	1836.5	5.50136	0.943681	784	1710.603
E3_M	45519	39470	2263.923	5.439708	0.902429	904	2090.667
E4_M	33500	29344	2074.347	5.758775	0.944772	893	2089.258
E5_M	41015	38294	1496.466	5.543533	0.934474	693	1432.598
E5_B	35252	31062	2159.01	5.761417	0.942031	914	2054.07
D4_B	34320	31196	2180.027	5.725533	0.936613	983	2032.103
E4_B	33474	29771	2026.333	5.36139	0.898783	930	2151.674
F4_B	35910	31203	2365.138	5.64385	0.920335	1011	2340.316
C5_B	43493	41145	1716.093	5.30059	0.916309	842	1751.972
D3_B	43373	38904	3287.923	5.811906	0.937047	1271	3198.089
F5_B	39770	35699	2206.984	5.659473	0.915611	1029	2149.622
C4_B	44572	42654	2147.087	5.610445	0.941063	951	2222.133
D5_B	38808	36313	2495.221	5.507001	0.930609	969	2200.325
E3_B	37264	32977	2531.567	5.800706	0.950721	1081	2588.977
