# Supplementary material for: Comparison of bacterial community structure and potential functions in hypoxic and non-hypoxic zones of the Changjiang Estuary
Source: PLoS One. 2019 Jun 6;14(6):e0217431. doi: 10.1371/journal.pone.0217431 (PMC6553723; doi:10.1371/journal.pone.0217431)
Supplement: S2 Table — Relative abundance (the mean values and standard deviations, std) of the major bacteria in the different water layers. An asterisk indicates a significant difference between the surface layer, the middle layer, and the bottom layer (ANOVA-test, P <0.05). (PDF) [file pone.0217431.s002.pdf]

S2 Table

OTU ID	S: mean. (%)	S: std. (%)	M: mean. (%)	M: std. (%)	B: mean. (%)	B: std. (%)	p-values
Balneolaceae	5.3385	5.5943	0.5492	0.6505	0.0914	0.0740	*0.0031
Cryomorphaceae	13.3433	5.3041	1.2190	0.5077	0.7299	0.3360	*0.0000
Erythrobacteraceae	4.0073	4.2394	0.1862	0.1254	0.0643	0.0362	*0.0025
Methylophilaceae	3.1661	1.8955	0.8008	0.5230	0.6417	0.3931	*0.0001
Nitrospinaceae	0.0575	0.1008	1.9129	1.3070	3.0337	1.3218	*0.0000
Pelagibacteraceae	24.4823	9.8956	45.4516	9.5455	43.8088	11.4615	*0.0002
Piscirickettsiaceae	0.2403	0.1990	4.2187	2.5648	5.8714	2.3867	*0.0000
Rhodobacteraceae	17.3074	3.6393	10.4072	2.8637	10.2673	3.5181	*0.0001
