# Supplementary material for: Comparison of bacterial community structure and potential functions in hypoxic and non-hypoxic zones of the Changjiang Estuary
Source: PLoS One. 2019 Jun 6;14(6):e0217431. doi: 10.1371/journal.pone.0217431 (PMC6553723; doi:10.1371/journal.pone.0217431)
Supplement: S3 Table — Simple parameters of different water layers in the Changjiang Estuary network. (PDF) [file pone.0217431.s003.pdf]

S3 Table

Parameters	Network (S)	Network (M)	Network (B)
Node	284	423	463
Edge	7603	12525	12583
Modularity	1.651	2.511	2.338
Number of communities	3	4	5
Average Degree	40.98	59.22	53.97
Average clustering coefficient	0.403	0.512	0.482
Average path length	2.19	2.15	2.23
Negative correlation	33%	42%	40%
