# Supplementary material for: Comparison of bacterial community structure and potential functions in hypoxic and non-hypoxic zones of the Changjiang Estuary
Source: PLoS One. 2019 Jun 6;14(6):e0217431. doi: 10.1371/journal.pone.0217431 (PMC6553723; doi:10.1371/journal.pone.0217431)
Supplement: S4 Table — Relative abundance (the mean values and standard deviations, std) of the functional genes in the different water layers. An asterisk indicates a significant difference between the surface layer, the middle layer and the bottom layer (ANOVA test, P <0.05). (PDF) [file pone.0217431.s004.pdf]

S4 Table

pathway	p-values (corrected)	B: mean (%)	B: std (%)	M: mean (%)	M: std (%)	S: mean (%)	S: std(%)
Folding, Sorting and Degradation	*0.00	2.71	0.04	2.66	0.05	2.51	0.04
Cell Growth and Death	*0.00	0.66	0.03	0.67	0.02	0.62	0.03
Circulatory System	*0.02	0.09	0.02	0.09	0.01	0.07	0.01
Digestive System	*0.00	0.04	0.01	0.03	0.00	0.05	0.01
Energy Metabolism	*0.00	6.68	0.22	6.71	0.18	6.33	0.21
Environmental Adaptation	*0.00	0.14	0.00	0.14	0.01	0.13	0.01
Enzyme Families	*0.04	1.82	0.05	1.79	0.03	1.77	0.03
Genetic Information Processing	*0.00	2.40	0.10	2.32	0.11	2.24	0.06
Lipid Metabolism	*0.00	3.87	0.05	3.90	0.05	4.09	0.08
Metabolism of Cofactors and Vitamins	*0.00	4.99	0.07	4.97	0.07	4.73	0.10
Metabolism of Other Amino Acids	*0.00	1.87	0.05	1.90	0.05	2.02	0.05
Metabolism of Terpenoids and Polyketides	*0.00	2.32	0.04	2.36	0.05	2.43	0.04
Nucleotide Metabolism	*0.00	3.85	0.06	3.83	0.06	3.70	0.08
Replication and Repair	*0.00	7.76	0.08	7.70	0.10	7.48	0.12
Signaling Molecules and Interaction	*0.00	0.09	0.02	0.09	0.01	0.15	0.02
Transcription	*0.00	1.99	0.07	1.99	0.06	2.19	0.08
Translation	*0.00	5.61	0.14	5.54	0.14	5.09	0.15
Transport and Catabolism	*0.00	0.29	0.02	0.29	0.01	0.33	0.02
Xenobiotics Biodegradation and Metabolism	*0.00	2.71	0.19	2.83	0.17	3.25	0.16

The symbol * represents the significance.
