# Supplementary material for: Comparison of bacterial community structure and potential functions in hypoxic and non-hypoxic zones of the Changjiang Estuary
Source: PLoS One. 2019 Jun 6;14(6):e0217431. doi: 10.1371/journal.pone.0217431 (PMC6553723; doi:10.1371/journal.pone.0217431)
Supplement: S3 Fig — The co-occurrence network of bacterial communities in the bottom layer. The edges represent correlation relationships. The nodes are sized by OTU betweenness and colored by phylum. (PDF) [file pone.0217431.s007.pdf]

S3 Fig

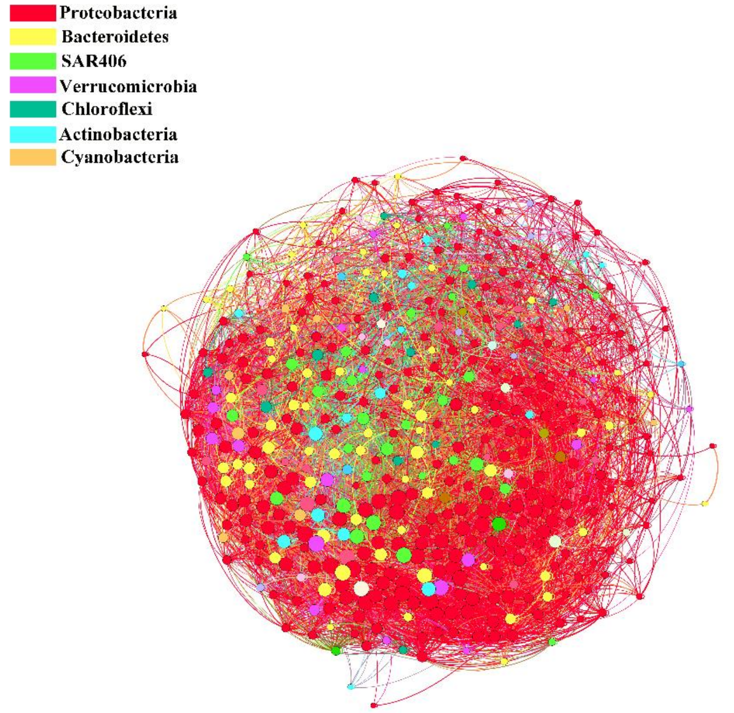
Proteobacteria
Bacteroidetes
SAR406
Verrucomicrobia
Chloroflexi
Actinobacteria
Cyanobacteria

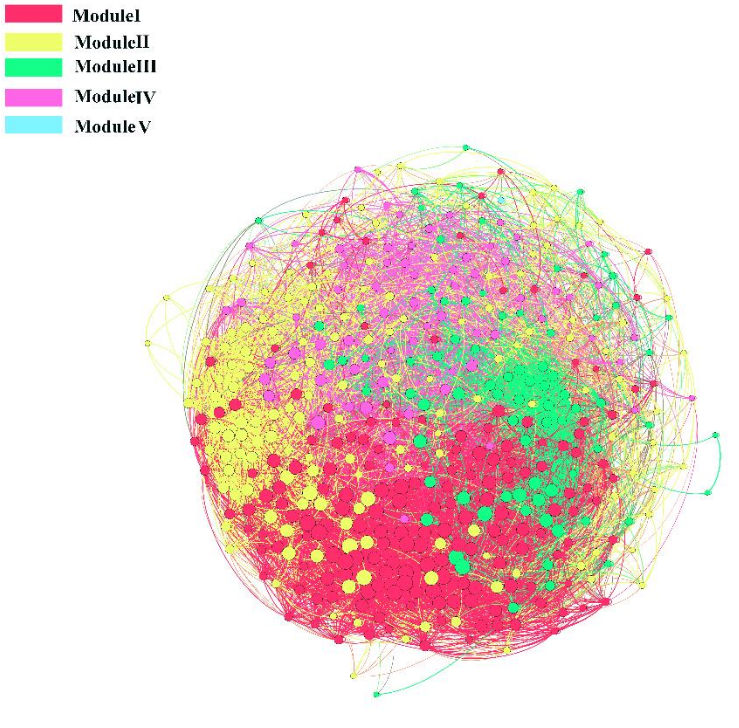
ModuleI
ModuleII
ModuleIII
ModuleIV
Module V
