# Supplementary figures and images for: Comparison of bacterial community structure and potential functions in hypoxic and non-hypoxic zones of the Changjiang Estuary
Source: PLoS One. 2019 Jun 6;14(6):e0217431. doi: 10.1371/journal.pone.0217431 (PMC6553723; doi:10.1371/journal.pone.0217431)

S4 Fig

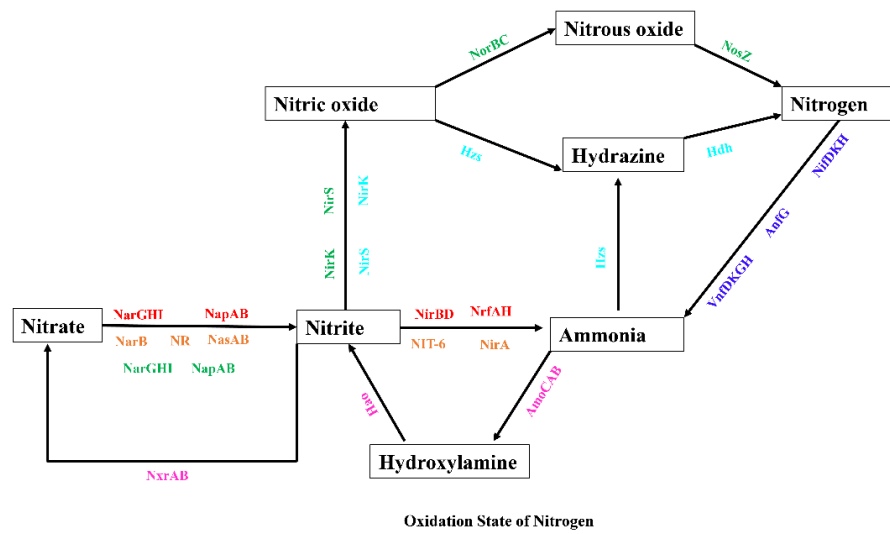

Supplement: S4 Fig — A map of the nitrogen cycle constructed using the KEGG nitrogen metabolism. It includes dissimilatory nitrate reduction, assimilatory nitrate reduction, denitrification, nitrogen fixation, nitrification and anammox. There are a variety of genes that encode enzymes that catalyze the important transformation reactions of various nitrogen forms ranging from oxidation states of +5 in nitrate to −3 in ammonium. (PDF) [file pone.0217431.s008.pdf]

S5 Fig

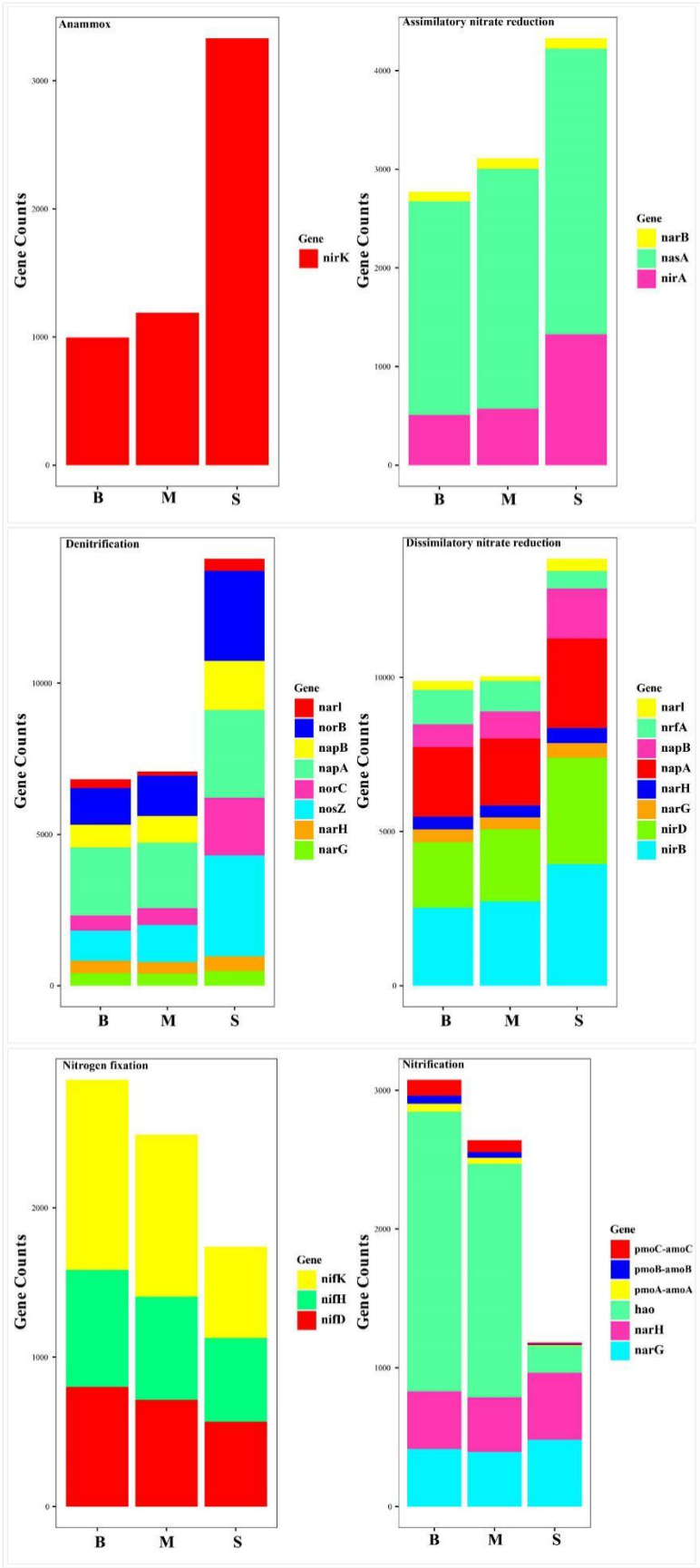

Supplement: S5 Fig — Relative abundance of the major categories of functional genes that encode the enzymes that catalyze nitrogen cycling pathways (dissimilatory nitrate reduction, assimilatory nitrate reduction, denitrification, nitrification, nitrogen fixation, and anammox) based on the KEGG database. (PDF) [file pone.0217431.s009.pdf]
